# The Social Utility of Ambivalence: Being Ambivalent on Controversial Issues Is Recognized as Competence

**DOI:** 10.3389/fpsyg.2018.00961

**Published:** 2018-06-22

**Authors:** Vincent Pillaud, Nicoletta Cavazza, Fabrizio Butera

**Affiliations:** ^1^Institut de Psychologie, Laboratoire de Psychologie Sociale, Université de Lausanne, Lausanne, Switzerland; ^2^Dipartimento di Comunicazione ed Economia, Università di Modena e Reggio Emilia, Modena, Italy

**Keywords:** ambivalence, attitudes, social value, judgment, controversy, warmth and competence

## Abstract

Research on attitudinal ambivalence is flourishing, but no research has studied how others perceive its expression. We tested the hypothesis that the expression of attitudinal ambivalence could be positively valued if it signals careful consideration of an issue. More specifically, ambivalence should be judged higher on social utility (competence) but not on social desirability (warmth), compared to clear-cut attitudes. This should be the case for controversial (vs. consensual) issues, where ambivalence can signal some competence. The participants in four experiments indeed evaluated ambivalence higher on a measure of social utility, compared to clear-cut (pro-normative and counter-normative) attitudes, when the attitude objects were controversial; they judged pro-normative attitudes higher for both social utility and social desirability when the attitude objects were consensual. Attitudinal ambivalence can therefore be positively valued, as it is perceived as competence when the expression of criticism is socially accepted.

## Introduction

It has been more than 40 years now since Scott (1968) and Kaplan (1972) introduced the concept of attitudinal ambivalence to describe the possibility that an individual could hold both positive and negative attitudes toward the same attitude object. Following their seminal work, an important body of research has been devoted to better understand the consequences of holding an ambivalent attitude toward a wide range of outcomes, from predicting behavior to well-being (e.g., Conner and Armitage, 2008). This work represents a very valuable research effort, as it appears that ambivalent attitudes are a widespread phenomenon (Basinger and Lavine, 2005; Weisbrode, 2012). Notwithstanding the importance of the phenomenon and the wealth of research that has investigated the consequences for those who hold ambivalent attitudes (for a recent review, see van Harreveld et al., 2015), only a limited number of studies have studied social perceptions of the expression of ambivalence, and no study has examined whether it could be viewed as having some social value. The present article aims to fill this gap in the literature by examining how individuals who express attitudinal ambivalence are judged in comparison with individuals who express univalent attitudes. More precisely, we hypothesized that ambivalence could be valued on controversial attitude objects in that pondering the pros and cons of an issue signals a competent judgment.

## Attitudinal ambivalence: strength or weakness?

Attitudinal ambivalence has primarily been studied as a dimension of attitude strength (Thompson et al., 1995; for a review, see Conner and Sparks, 2002). In comparison with univalent attitudes, ambivalent attitudes have been shown to be less stable over time, more pliable, less likely to guide information processing, and less predictive of intentions and further behavior (Armitage and Conner, 2004). In this perspective, ambivalent attitudes are presented as weak attitudes (Sawicki et al., 2013). Furthermore, as ambivalence is derived from mixed attitudes that rely on conflict and indecision, it has often been associated with cognitive dissonance (Priester and Petty, 1996; Clark et al., 2008; van Harreveld et al., 2009a). Consequently, ambivalent individuals who experience cognitive dissonance have been described as willing to “solve their ambivalence” (Festinger, 1957; Newby-Clark et al., 2002). In line with this view, holding an ambivalent attitude has been considered as an aversive state (Nordgren et al., 2006) that potentially generates psychological discomfort in decision-making situations (van Harreveld et al., 2009b). Thus, the results above, combined with the finding that ambivalent individuals seem to be motivated to reduce their ambivalence (e.g., Bell and Esses, 1997, 2002), appear to suggest that the expression of an ambivalent attitude may be viewed negatively. However, other lines of research have portrayed it in a more positive light.

Mixed attitudes can also lead to benefits (Rothman et al., 2017). If attitudinal ambivalence is generally known to be vulnerable to influence, as discussed above, it can increase the likelihood of changing some specific behaviors for the better (Armitage and Conner, 2000; Armitage and Arden, 2007). For example, Lipkus et al. (2005) reported that attitudinal ambivalence was positively linked to the desire to quit smoking among teen smokers. Likewise, the intention to eat junk food decreased more for ambivalent individuals compared to univalent individuals when confronted with a negatively framed persuasive message (Yan, 2015). Ambivalence can then be seen as adaptive when it focuses on contributing to changing negative behaviors. It can also provide advantages in intergroup relations. For instance, Pagliaro et al. (2012) investigated how low-status individuals can cope with social identity threats (namely, when Italians were disadvantaged compared to the British) as a function of their identification with their ingroup. They found that low-identifiers, as opposed to high-identifiers, perceived their ingroup as more variable (consisting of various positive and negative traits rather than only positive traits). Interestingly, these authors interpreted such attitudes as an adaptive form of social creativity in the face of identity threat. Finally, Costarelli (2011) described ambivalence toward out-groups as a more “defensible, balanced, and realistic reaction” to the relevant stereotypic traits of the out-group members (pp. 51–52). The reasoning behind this statement is that endorsing an ambivalent attitude allows the individuals to express negative attitudes that fulfill their “need to be prejudiced” toward out-group members while simultaneously expressing positive attitudes, which results in a balanced position that might be positively perceived by others. Therefore, some recent research has suggested that the expression of ambivalent attitudes could also be positively perceived.

## The social value of attitudinal ambivalence

Some attitude objects are more likely to arouse ambivalence compared to others (Conner et al., 1998; Dahl et al., 2005). For example, Breckler (2004) reported that 41.1% of the students were ambivalent on nuclear power whereas only 11% were ambivalent about legalized abortion and 15.1% about gun control laws. Such different ratios could be the result of the salience of the social norm associated with the attitude objects. When there is a clear social norm (consensus), an ambivalent attitude could then be seen as a deviant attitude in comparison with the socially valued (univalent) one. Subsequently, it might be perceived less positively and expressed less frequently. Conversely, when there is a strong debate and competing arguments regarding an issue, holding a certain degree of ambivalence could be positively valued, as it might signal that one is aware of the complexity of the issue.

Thus, as Maio and Haddock (2004, 2009) suggested, ambivalent attitudes toward controversial issues could be valued. Indeed, recent research by Pillaud et al. (2013) provides direct support for this reasoning. These authors reported that individuals displayed a more ambivalent attitude when they had to present themselves in a positive way, and they did so especially when they had to express themselves on a controversial attitude object (e.g., Genetically Modified Organisms), as opposed to a consensual attitude object (e.g., tooth brushing). In sum, contrary to a longstanding tradition that views attitudinal ambivalence as a weak form of attitudes and an aversive state, recent research has revealed that individuals could express ambivalent attitudes when they want to be positively evaluated, to the extent that the attitude object is controversial.

## Why can attitudinal ambivalence provide value?

Why can one be positively valued when expressing ambivalent attitudes? In the reasoning presented above, we suggested that expressing ambivalence when treating a controversial issue might signal that one is pondering the pros and the cons, which might imply some sort of competence. However, to date, no research has demonstrated that attitudinal ambivalence is associated with competence. Despite the lack of direct evidence, literature on social judgment provides important insights for this hypothesis. Indeed, a well-established line of work has shown that people's judgments about individuals, groups (stereotypes), and objects tend to be organized according to two main dimensions (Kervyn et al., 2010), namely warmth and competence.

These two dimensions have been variously termed, for instance, value vs. dynamism (Osgood, 1962), social desirability vs. intellectual desirability (Rosenberg et al., 1968), self-profitability vs. other-profitability (Peeters, 1992), communion vs. agency (Abele and Wojciszke, 2007), warmth vs. competence (Fiske et al., 2002), and social desirability vs. social utility (Dubois and Beauvois, 2005). If these different terms cover similar concepts, they lead to different approaches in the study of the social judgment (Beauvois and Dubois, 2009). The approach proposed by Beauvois and Dubois (Beauvois, 1995; Dubois and Beauvois, 2005) better fits the general purpose of the present research, considering that social desirability and social utility refer to the “social worth” conveyed by individuals during an evaluative process (Beauvois and Dubois, 2009). In the present research, we want to study precisely why individuals expressing attitudinal ambivalence can be valued. Social desirability corresponds to the individuals' ability to obtain a positive evaluation from others; in this respect, social desirability is the societal-level equivalent of a judgment of warmth. Conversely, social utility corresponds to the ability to satisfy the requirements of a given social environment and thus to the individuals' chances of success in social life (Dubois and Beauvois, 2005; Darnon et al., 2009). In this respect, social utility is the societal-level equivalent of a judgment of competence. Based on this distinction, it is then possible to predict that attitudinal ambivalence should be valued in terms of social utility but not necessarily in terms of social desirability.

An analysis of the literature on the effects of attitudinal ambivalence provides some indirect support for this idea. On the one hand, research in which ambivalence has been studied for its higher proclivity to be influenced by others could be linked to social desirability. In particular, some research by Bell and Esses (1997, 2002) is in line with this reasoning. In their first study, the researchers reported that positive and negative information influence ambivalent individuals more than non-ambivalent individuals (i.e., greater response amplification). Ambivalent participants more strongly endorsed a positive/negative attitude after the presentation of positive/negative information. More interestingly, response amplification was further related to social desirability concerns in that it was greater when ambivalence was described negatively rather than positively (Bell and Esses, 2002, Study 2). In other words, individuals complied with the message that presented ambivalence negatively. In support of this reasoning, Cavazza and Butera (2008) reported that more ambivalent individuals purposely sided with a normative source of influence on topics that were directly related to the source's message but not on topics more indirectly related to this message, unlike less ambivalent individuals. Thus, the proclivity of ambivalent individuals to be influenced—quite a frequent phenomenon (see Armitage and Conner, 2000; Hodson et al., 2001 for instance)—might therefore be a sign of the attempt to avoid the social undesirability of expressing ambivalent attitudes.

On the other hand, the aforementioned research by Cavazza and Butera (2008) also suggested that attitudinal ambivalence may be linked to social utility, as it may be instrumental in adapting to the social environment, for instance, to resisting persuasive pressure of the majority, since ambivalent participants comply at a direct but not indirect level. Similarly, Weick (2002, 2004), in his theoretical papers, approached the concept of ambivalence as being an “optimal compromise.” Ambivalence was conceived of as a wise attitude that allows equilibrium between knowledge and doubts (Weick, 1998, 2004). Indeed, in a changing environment (such as fire crews fighting fires for instance), competing tendencies can prevent people from unwarranted persistence of behavior while providing both confidence and cautiousness. In other terms, ambivalence can be perceived as helpful in that it can allow a fast adaptation to varying situations. Ambivalence has also been shown to be beneficial in helping individuals interpret complex situations (Guarana and Hernandez, 2015). In line with this idea, it also has been suggested that ambivalence could be linked with critical thinking, namely by enhancing mindfulness (Fiol and O'Connor, 2003) or cognitive elaboration (Yan, 2015). For instance, Piderit (2000) argued that the expression of ambivalence is more likely to generate dialogue in comparison with the expression of support or opposition. Likewise, Green et al. (2000) reported that the coexistence of conflicting attitudes could lead to higher message scrutiny and integrative complexity. Erisen and Erisen (2014) also highlighted that ambivalence could lead to greater tolerance of different perspectives, resulting, for example in their study, in greater support for Turkish membership in Europe. Focusing on creativity, Fong (2006) reported that emotional ambivalence could lead individuals to make more associations (i.e., to be more creative) in comparison with happy or sad participants. Ambivalent individuals were also found to be more accurate in their judgment and forecasting compared to individuals experiencing single-affect states (Rees et al., 2013). Finally, ambivalence toward Europe was found to be positively related with objective knowledge and news media consumption (i.e., political sophistication; Stoeckel, 2013). Attitudinal ambivalence could therefore be perceived as a form of competence and subsequently be perceived as socially useful.

If attitudinal ambivalence were associated with competence, it still needs to be determined whether attitudinal ambivalence could be associated with competence in case of controversial issues, as proposed above, and in which cases. Stoeckel (2013), who reported that disagreement among the elites (i.e., disagreement among political parties and leaders) on the attitude toward Europe did increase the citizens' probability of being ambivalent by 16.5%, indirectly supported this idea. The reasoning is that if elites are divided on an issue, their positions will be more differentiated and more likely to be in competition with one another. In return, citizens would be more likely to be ambivalent toward the issue. Similarly, Plambeck and Weber (2010) observed that strategic ambidexterity in an organization (i.e., the inclusion of both offensive and defensive strategies) leads to more ambivalence in a decision-making situation. In a similar vein, Keele and Wolak (2008) highlighted that periods of election campaigns could also represent a source of ambivalence in the population, since elections generate a debate between different political sides; hence, different points of view are salient in people's environment. Indeed, as Larsen pointed out, ambivalence can indicate that one is “coming to grips with the complexity of the world” (Larsen, cited in Wang, 2010).

In sum, the vast majority of research to date in social psychology sustains that attitudinal ambivalence is a weak form of attitude and an aversive state; in other words, a form of attitude that should not be valued by individuals. However, some recent research (in social psychology and in other research areas) has shown that attitudinal ambivalence may have a positive effect (such as enhancing mindfulness, wisdom, or creativity) and allow resisting social influence. Four experiments have been designed to investigate whether expressing attitudinal ambivalence in case of controversial issues does indeed generate a positive image of the target in observers, and if it does, why.

## Hypotheses and overview

Based on the reasoning presented in the previous section, we hypothesized that the expression of ambivalence should be judged higher on social utility in comparison with univalent attitudes when expressing ambivalence toward *controversial* attitude objects (Hypothesis 1a). This should not be the case for social desirability. Indeed, social norms (what majorities or relevant others say and do) and social desirability are often associated in the literature, since social norms exert social influence (Gilibert and Cambon, 2003; Johnson and van de Vijver, 2003). In other words, social desirability is related to the extent to which an attitude expresses approval or disapproval of a topic that is widely approved or disapproved. As noted above, we view ambivalent attitudes as potentially indicating competence, that is, a trait related to succeeding in a system rather than pleasing the members of this system. We thus reasoned that ambivalent attitudes cannot be judged higher on social desirability compared to pro-normative attitudes. Instead, we hypothesized that we should observe a linear effect of the displayed attitude on social desirability in that the pro-normative attitude should be evaluated as the most desirable, ambivalence should be valued less (as it deviates from the pro-normative attitude), and the counter-normative attitude should be the least valued (as it is the most deviant attitude) (Hypothesis 1b).

As non-controversial *consensual* attitude objects imply that there is a strong consensus on what the expressed response should be (Pérez and Mugny, 1996), we reasoned that the pro-normative attitude should be evaluated the highest, regardless of the judgment's dimension. Thus, we hypothesized that pro-normative attitudes should be evaluated as higher on social utility compared to the ambivalent ones, which should in turn be evaluated as more useful compared to the counter-normative one (Hypothesis 2a). We expected to find the same linear effect on social desirability (Hypothesis 2b).

To test our hypotheses, we used the so-called “judge paradigm” (Jellison and Green, 1981; Dubois, 1988). This paradigm has been widely used to study the value of psychological constructs. For instance, it has been successfully used to investigate the social value of the preference for consistency (Channouf and Mangard, 1997; Sénémeaud et al., 2011), of intrinsic motivation (Cassignol-Bertrand et al., 2006), of the belief in a just world (Alves and Correia, 2010), of individualism and collectivism (Dubois and Beauvois, 2005; Green, 2006), of achievement goals (Darnon et al., 2009), and of organizational citizenship behavior (Esnard and Jouffre, 2008). In one of the versions of this paradigm, the participants were invited to evaluate several targets who vary in their endorsement of the investigated trait (see Cambon et al., 2006). In our research, we presented three profiles of three alleged previous participants (namely, a bogus attitude questionnaire supposedly filled out by the target), presenting a target with a positive attitude, one who holds a negative attitude, and one displaying ambivalence. The participants were required to judge each profile on both social desirability and social utility.

We tested the first two hypotheses on different attitude objects (Experiments 1–4). More precisely, Experiments 1 and 2 tested Hypothesis 1 on controversial attitude objects (immigration and death penalty), and Experiments 3 and 4 tested Hypothesis 2 on non-controversial attitude objects (organic products and recycling).

The level of controversy over the above four attitude objects has been assessed in a pilot study with a sample of Swiss students, which was comparable to that of the main experiments. Furthermore, an analysis of the literature revealed whether positive or negative attitudes are pro or counter-normative; this may be evident for consensual issues but needs to be studied for controversial issues. Concerning controversial issues, studies have found that although the issue of immigration is recognized as controversial, Swiss citizens are on average in favor of new immigration and hence tend to hold a positive attitude toward it (Meuleman et al., 2009). Moreover, like most Europeans, Swiss citizens held predominantly negative attitudes toward death penalty (Unnever, 2010). Regarding consensual issues, several papers convincingly highlighted the social normativeness of holding a positive attitude toward recycling and pro-environmental behaviors (e.g., Turaga et al., 2010; Schwab et al., 2014). This effect was also observed for organic products (Aertsens et al., 2009), toward which most consumers held a positive attitude (Saba and Messina, 2003; Kihlberg and Risvik, 2007).

## Pilot study

We conducted a first pilot study to ascertain that immigration and death penalty are indeed perceived as controversial issues and that organic products and recycling are considered non-controversial issues. We selected a sample comparable to the sample of participants recruited in the main experiments.

### Participants and method

One hundred and twenty-one students from a medium-size Swiss university volunteered in this pilot study conducted on the Internet using LimeSurvey. Among several other attitude objects, the participants were asked to indicate their perception of controversy or consensus over immigration, death penalty, organic products, and recycling. More precisely, they were asked to complete three 7-point bipolar scales devised for this pilot study for each attitude object. The first bipolar scale ranged from *consensus* (1) to *controversy* (7), the second from *no debate* (1) to *debate* (7), and the third from *a mutual agreement* (1) to *a polemic disagreement* (7). The Cronbach's alphas ranged from 0.75 for immigration to 0.90 for death penalty; therefore, we averaged the answers provided on the three items for each attitude object and considered them our dependent variables. The scale ranged from 1 (*perception of consensus*) to 7 (*perception of controversy*).

### Results and discussion

To test whether these attitude objects should be perceived as controversial or consensual, we conducted *t*-tests against the mid-point of the scale (i.e., 4). The analyses revealed that immigration and death penalty were evaluated as being controversial, (*M* = 5.92, *SD* = 0.90), *t*_(120)_ = 23.52, *p* < 0.001, $\eta_{\text{p}}^{2}$ = 0.82 for immigration, and (*M* = 4.38, *SD* = 1.88), *t*_(120)_ = 2.20, *p* = 0.030, $\eta_{\text{p}}^{2}$ = 0.03 for death penalty. Moreover, organic products and recycling were evaluated as consensual, (*M* = 3.26, *SD* = 1.22), *t*_(120)_ = −6.69, *p* < 0.001, $\eta_{\text{p}}^{2}$ = 0.27 for organic products, and (*M* = 3.20, *SD* = 1.65), *t*_(120)_ = −5.31, *p* < 0.001, $\eta_{\text{p}}^{2}$ = 0.19 for recycling.

## Experiment 1

### Method

#### Participants and design

This web-based experiment was run on LimeSurvey. The students of a medium-size Swiss university were solicited to participate in the experiment as a part of psychology tutorials. Fifty-two participants (34 females, 18 males) completed the questionnaire. Their mean age was 22.06 years (*SD* = 2.91). Preliminary analyses revealed that sex and age had no effect on the studied outcomes (all *ps* > 0.1). The type of attitude profiles was included as a within-participants variable presented in a random order. The participants had to evaluate three alleged previous participants who expressed a positive attitude, a negative attitude, and an ambivalent attitude toward the same attitude object. No order effect was observed on the evaluations (all *ps* > 0.1). Therefore, order of presentation, sex and age have not been included in the reported analyses. This is true for all other experiments.

#### Procedure and materials

The experiment was presented as a follow-up of a previous study on attitudes. In this bogus previous study, the participants were allegedly asked to complete eight items assessing their attitude toward immigration measured on a 7-point Likert scale (ranging from 1 = *not at all* to 7 = *very much*). In fact, the experimenter had filled out the questionnaire in advance and generated three specific profiles of participants. The first profile displayed a univalent positive attitude toward immigration (positive condition) by always circling either 6 or 7. The second profile reported a univalent negative attitude (negative condition) by only circling 1 or 2. The third profile presented an ambivalent attitude by agreeing on four of the items (circling 6 or 7) and disagreeing on the four others (circling 1 or 2). This profile constituted the ambivalent condition. It is important to note that in the ambivalent profile, the target is depicted as holding both strongly positive and strongly negative attitudes toward the same issue on different dimensions, which implies that the target is ambivalent rather than does not care about or holds moderate attitudes toward the issue. These dimensions were not explicitly emphasized to avoid suspicion. Nevertheless, the participants could realize that the target strongly agreed with items 1–4 and strongly disagreed with items 5–8, based on which they could infer that these items referred to different aspects of the same topic (e.g., ethical and policy aspects of immigration; see Appendix in Supplementary Material).

The participants were then asked to evaluate each profile based on the answers to 8 items, with 4 items measuring social desirability (nice, likable, appreciable and pleasant) and 4 items assessing social utility (competent, smart, gifted and likely to succeed academically), using the same materials as Dompnier et al. (2008). Evaluations were made on a 7-points Likert scale ranging from 1 (*not at all*) to 7 (*very much*) for each of these traits. The Cronbach's alphas for social desirability ranged from 0.96 to 0.98, depending on the profile. The alphas for social utility varied from 0.94 to 0.97. Therefore, we averaged the four traits for each dimension and used them as dependent variables^1^.

### Results and discussion

Since immigration has been shown in the pilot study to be a controversial attitude object, we hypothesized that the ambivalent profile should be rated the highest on social utility in comparison with the two other profiles (H1a). On the other hand, given that on average Swiss individuals are open to new immigration (Meuleman et al., 2009), we hypothesized that we should observe a decreasing linear trend on social desirability from the positive to the ambivalent and to the negative profiles (H1b).

#### Social utility

To test hypothesis 1a, we designed a within-participant planned contrast, testing the score in the ambivalent condition (2) against both the score in the positive (−1) and in the negative condition (−1). Moreover, the score in the positive condition (1) was tested against the score in the negative condition (−1) in an orthogonal contrast; the score in the ambivalence condition was set as 0. The proper use of contrast analysis requires the planned contrast that tests the hypothesis to be significant, and the orthogonal contrast testing the residual to be non-significant (Judd and McClelland, 1989).

The test of this planned contrast reached significance, *F*_(1, 51)_ = 4.93, *p* = 0.031, $\eta_{\text{p}}^{2}$ = 0.08. In comparison with both the positive (*M* = 4.00, *SD* = 1.19) and the negative (*M* = 3.75, *SD* = 1.37), the ambivalent profile (*M* = 4.31, *SD* = 1.40) was evaluated as significantly higher on social utility. The orthogonal contrast was not significant, *F* < 1.

#### Social desirability

To test hypothesis 1b on this dimension, we designed a within-participant planned contrast whereby the score in the positive condition (1) has been tested against the score in the negative condition (−1) and the score in the ambivalent condition was set as 0 to test the expected linear relation. Moreover, the score in the ambivalence condition (2) was tested against the score in the negative (−1) and in the positive condition (−1) in an orthogonal contrast.

**Figure 1:**
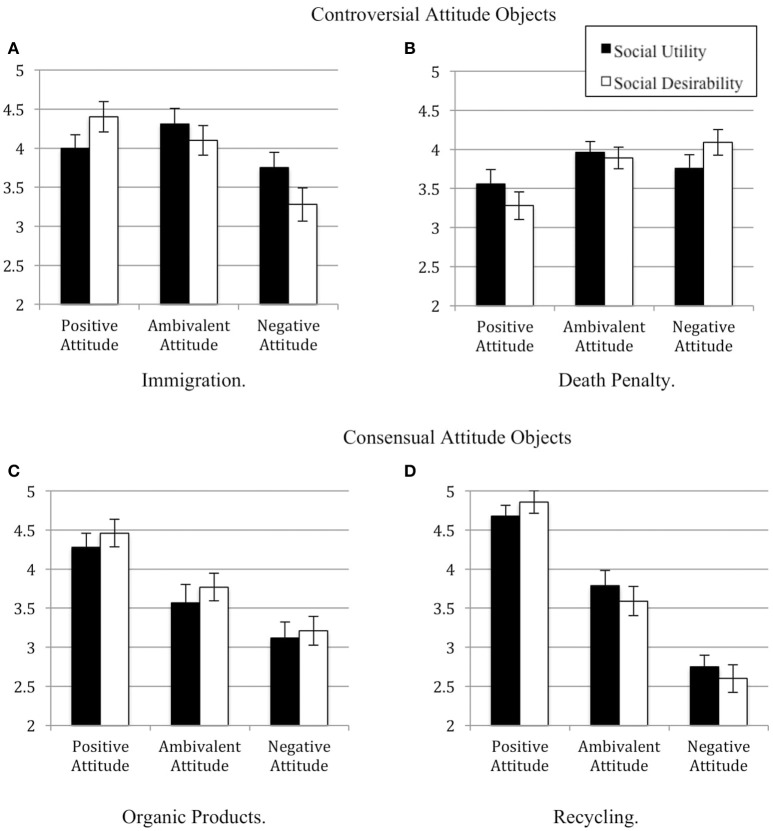
Social utility and social desirability as a function of the profiles' attitude and the controversy level of the attitude object. **(A)** Immigration. **(B)** Death penalty. **(C)** Organic products. **(D)** Recycling.

The test of our planned linear contrast yielded a significant effect on social desirability, *F*_(1, 51)_ = 12.44, *p* < 0.001, $\eta_{\text{p}}^{2}$ = 0.19. Being in favor of immigration was rated as more desirable (*M* = 4.40, *SD* = 1.37) than being ambivalent (*M* = 4.10, *SD* = 1.35), than being against immigration (*M* = 3.28, *SD* = 1.50). The test of the orthogonal contrast did not reach significance, *F*_(1, 51)_ = 2.43, *p* > 0.10. These results provide support for our first hypothesis, in that the participants rated the ambivalent profile the highest on social utility, whereas we observed a linear decrease in social desirability moving from the positive attitude to the negative one (see Figure 1A).

## Experiment 2

### Method

#### Participants and design

This web-based experiment was run on LimeSurvey, administered through social network websites, and tested the same hypotheses as those proposed in Experiment 1. The participants were recruited to participate in an experiment about death penalty, again a controversial issue according to the pilot study. Sixty-nine participants completed the questionnaire. Their mean age was 23.96 years (*SD* = 6.55). The design was identical to that of Experiment 1. Four participants were dropped from the analysis because of a large Cook's distance (Hutcheson and Sofroniou, 1999). The final sample consisted of 45 females and 20 males. Their mean age was 24.08 years (*SD* = 6.72).

#### Procedure and materials

The experiment was introduced as a study about attitudes toward death penalty. The same procedure was used as in Experiment 1, that is, the participants had to judge three alleged participants of a previous study. Profiles displaying a positive attitude, an ambivalent attitude, and a negative attitude were presented in a random order to the participants who had to evaluate each profile with the same four social desirability and four social utility traits as before. The Cronbach's alphas for social desirability ranged from 0.95 to 0.97, depending on the profile, the alphas for social utility varied from 0.92 to 0.95. Therefore, we averaged the four traits for each dimension and considered these variables as our main dependent variables.

### Results and discussion

Since death penalty has been shown in the pilot study to be a controversial attitude object, we hypothesized that the ambivalent profile should be rated the highest on social utility in comparison with the two other profiles (H1a). On the other hand, given that most Swiss individuals are against death penalty (Unnever, 2010), we hypothesized that we should observe a decreasing linear trend on social desirability across the positive, the ambivalent, and the negative profiles.

#### Social utility

The score in the ambivalent condition (2) has been tested against both the score in the positive (−1) and in the negative condition (−1). The test of this planned contrast (H1a) yielded a significant effect, *F*_(1, 64)_ = 4.74, *p* = 0.033, $\eta_{\text{p}}^{2}$ = 0.06. In comparison with both the positive (*M* = 3.56, *SD* = 1.44) and the negative (*M* = 3.76, *SD* = 1.36), the ambivalent profile (*M* = 3.96, *SD* = 1.11) was evaluated the highest on social utility. The positive profile and the negative profile, compared in the orthogonal contrast, did not differ significantly, *F* < 1.

#### Social desirability

The test of a linear contrast (H1b) yielded a significant effect on social desirability, *F*_(1, 64)_ = 8.33, *p* = 0.005, $\eta_{\text{p}}^{2}$ = 0.11. Being against death penalty was rated as more desirable (*M* = 4.09, *SD* = 1.30) compared to being ambivalent (*M* = 3.89, *SD* = 1.11) or favorable (*M* = 3.28, *SD* = 1.42). The test of the orthogonal contrast did not reach significance, *F*_(1, 68)_ = 2.65, *p* > 0.10. These results provide support for our first hypothesis with a different controversial attitude object. Specifically, the participants rated the ambivalent profile the highest on social utility, whereas we observed a linear decrease in desirability from the negative attitude to the positive one (see Figure 1B).

## Experiment 3

### Method

#### Participants and design

This web-based experiment was run on LimeSurvey to test Hypothesis 2. This time, the participants were recruited through social network websites to participate in an experiment about organic products, a non-controversial attitude object. Thirty-five participants (28 females, 7 males) completed the questionnaires. Their mean age was 29.49 years (*SD* = 16.38).

#### Procedure and materials

The experiment was introduced as a study about attitudes toward organic products. The same procedure was used as for previous experiments: Profiles displaying a positive attitude, an ambivalent attitude, and a negative attitude were presented in random order to the participants who had to evaluate each profile with the same four social desirability and four social utility traits as before. The Cronbach's alpha across profiles ranged from 0.96 to 0.98 for social desirability and from 0.89 to 0.97 for social utility.

### Results and discussion

#### Social utility

In Hypothesis 2, we predicted that for non-controversial attitude objects, we should observe a linear trend for both social utility and social desirability. Consequently, the score of social utility should be the highest for the positive profile, indicating that a positive attitude toward organic products is pro-normative (Aertsens et al., 2009), and the lowest for the negative profile, with the ambivalent profile in the middle. Contrast analyses revealed that the linear contrast was indeed significant, *F*_(1, 34)_ = 22.68, *p* < 0.001, $\eta_{\text{p}}^{2}$ = 0.40. The participants evaluated the positive profile the highest (*M* = 4.28, *SD* = 1.03), followed by the ambivalent profile (*M* = 3.57, *SD* = 1.37) and the negative profile (*M* = 3.12, *SD* = 1.17). The orthogonal contrast did not reach significance, *F* < 1.

#### Social desirability

The test of a linear contrast yielded a significant effect, *F*_(1, 34)_ = 31.93, *p* < 0.001, $\eta_{\text{p}}^{2}$ = 0.48. Being in favor of organic products was judged as being more desirable (*M* = 4.45, *SD* = 1.03) in comparison with an ambivalent (*M* = 3.77, *SD* = 1.02) and a negative profile (*M* = 3.21, *SD* = 1.06). The orthogonal contrast did not reach significance, *F* < 1.

As predicted by Hypotheses 2a and 2b, the positive profile was rated the highest on both social desirability and social utility, followed by the ambivalent and the negative profiles (see Figure 1C).

## Experiment 4

### Method

#### Participants and design

This web-based experiment was run on LimeSurvey again to test Hypothesis 2. This time, the participants were recruited through social network websites to participate in an experiment about recycling, a non-controversial attitude object. Fifty-one participants (38 females, 13 males) completed the questionnaires. Their mean age was 25.39 years (*SD* = 10.08).

#### Procedure and materials

The participants were told that we were conducting a study about attitudes toward recycling. The same procedure was used as in previous experiments. Three profiles displaying a positive attitude, an ambivalent attitude, and a negative attitude were presented in a random order to the participants who had to evaluate each profile with the same four social desirability and four social utility traits as before. The Cronbach's alpha for social desirability ranged from 0.92 to 0.96 while alphas for social utility varied from 0.83 to 0.82. Therefore, we calculated the means of the four traits for each dimension and used them as our dependent variables.

### Results and discussion

#### Social utility

We hypothesized a linear trend on both social utility and social desirability, as recycling emerged as a non-controversial topic in the Pilot Study, with the positive attitude being the most positively evaluated, since social norms encourage pro-environmental behaviors (Turaga et al., 2010; Schwab et al., 2014). The test of a linear contrast yielded a significant effect, *F*_(1, 50)_ = 67.04, *p* < 0.001, $\eta_{\text{p}}^{2}$ = 0.57. Being in favor of recycling was judged as more useful (*M* = 4.68, *SD* = 0.97) in comparison with an ambivalent profile (*M* = 3.79, *SD* = 1.38), which in turn was judged more useful compared to a negative profile (*M* = 2.75, *SD* = 1.05). The orthogonal contrast was not significant, *F* < 1.

#### Social desirability

The test of a linear contrast yielded a significant effect, *F*_(1, 50)_ = 64.23, *p* < 0.001, $\eta_{\text{p}}^{2}$ = 0.56. The profile presenting a positive attitude was evaluated more desirable (*M* = 4.86, *SD* = 1.04) compared to the ambivalent profile (*M* = 3.59, *SD* = 1.32), which in turn was evaluated as more desirable compared to the negative profile (*M* = 2.61, *SD* = 1.25). The orthogonal contrast did not reach significance, *F* < 1. Concerning organic products, the positive pro-normative profile was rated the highest on both social utility and social desirability, followed by the ambivalent and the negative profile (see Figure 1D).

## Meta-analysis

In the work reported above, we used four different attitude objects in four separate studies. In order to ensure that our effects on social utility were driven by the type of the attitude object (i.e., controversial vs. consensual), as proposed, and not by the attitude object itself (death penalty or recycling, for instance), the data of the four studies have been combined into a new database to run a small-scale meta-analysis (Hox et al., 2017). This analysis has not been conducted for social desirability, as the effects were not expected to differ as a function of the type of attitude object. We built a three-level multilevel analysis in which social utility scores (*N*_1−4_ = 621 level-1 units, i.e., 207 participants who had three social utility scores, one per profile: Study 1 = 52^*^3, Study 2 = 69^*^3, Study 3 = 35^*^3 and Study 4 = 51^*^3) were nested within participants (*K*_1−4_ = 207 level-2 units), which were nested within studies (*L*_1−4_ = 4 level-3 units, random effect). Thus, the intercept was allowed to vary across both participants and studies. All participants were included in the analysis (removing the four outliers in Study 2 did not alter any of the reported results).

### Results and discussion

The first analysis tested whether social utility judgments significantly differed as a function of type and profiles. Random effects pertaining to participants and studies were also included in the model. The 2 (type of attitude object: controversial, consensual) × 3 (profile: positive, negative, ambivalent) interaction reached significance, $\chi_{\left(2,\;621\right)}^{2}$ = 33.35, *p* < 0.001. Ratings therefore differed as a function of the type of attitude object and profile.

The interaction has then been decomposed into two simple effects to reproduce the analyses carried out in experiments 1–4: Attitude type was then dummy coded (controversial, consensual) and the same two contrasts used in the single experiments were tested, i.e., a quadratic contrast (−1 2 −1) and a linear contrast (1 0 −1) for the pro-normative, ambivalent and counter-normative condition. The effect of type of attitudes object was not significant, *Z* = 1.53, *p* = 0.126. Contrast analyses on controversial attitude objects revealed a significant effect of the quadratic contrast on social utility, *Z* = 2.57, *p* = 0.01 whereas the linear contrast was not significant, *Z* = −1.45, *p* = 0.147. Conversely, the analysis on consensual attitude objects revealed that the linear contrast reached significance, *Z* = −8.44, *p* < 0.001 whereas the quadratic did not, *Z* = −0.04, *p* = 0.965.

These results thus reproduced those observed in experiments 1–4 and suggest the moderating role of controversial/non-controversial attitude objects. Ambivalent attitudes were valued more in terms of social utility compared to the expression of both a pro-normative and a counter-normative attitude when the topic was controversial; however, a pro-normative attitude was favored when the topic was non-controversial.

## General discussion

The vast majority of research on attitudes to date considers that attitudinal ambivalence is a weak form of attitude and an aversive state; in other words, a form of attitude that individuals should not value given its consequences. However, this speculation has hitherto never been tested and no research has assessed the social perception of the expression of ambivalence. Two elements make this research essential. First, the fact that individuals display ambivalence in an attempt to achieve positive self-presentation (Pillaud et al., 2013), although interesting in and of itself, does not imply that such attempt is successful and that the display of ambivalent attitudes is indeed socially valued by an observer (Dubois and Beauvois, 2005). Second, as we noted above, although some theoretical analyses have suggested that ambivalence may be valued, no research so far has provided empirical evidence that this may be the case or investigated why expressing attitudinal ambivalence may generate a positive image in observers. In fact, no work has directly investigated the social perception of the expression of ambivalent attitudes.

In this article, we proposed that ambivalence could have a specific function of signaling competence when being expressed on controversial issues. This reasoning has been rooted in several studies that linked ambivalence and a wide array of cognitions, for instance, critical thinking and wisdom (Weick, 1998, 2004; Piderit, 2000), amount of knowledge (Stoeckel, 2013), and strategic thinking (Plambeck and Weber, 2010). Furthermore, ambivalence has been shown to serve some adaptive function (Guarana and Hernandez, 2015). Indeed, ambivalent attitudes were discussed as providing equilibrium in complex situations (Costarelli, 2011; Pagliaro et al., 2012) and allowing individuals to resist majority influence (Cavazza and Butera, 2008). Thus, we hypothesized that individuals expressing ambivalent attitudes toward controversial issues could be valued on social utility, unlike individuals expressing univalent attitudes.

More specifically, we hypothesized that an individual expressing ambivalent attitudes on controversial attitude objects should be judged higher on social utility in comparison with an individual expressing univalent attitudes, either pro-normative or counter-normative (Hypothesis 1a). We consequently predicted that the benefit of expressing ambivalent attitudes should not be observed on social desirability. We expected to observe a decreasing linear effect from the pro-normative to the ambivalent attitude, with the counter-normative attitude being less desirable (Hypothesis 1b). Because consensual attitude objects imply a strong consensus about what the expressed response should be (Pérez and Mugny, 1996), we hypothesized that the pro-normative attitude should be rated the highest for both social utility and social desirability. Hence, we predicted that they should be evaluated as more useful compared to the ambivalent one, which should in turn be evaluated as more useful compared to the counter-normative one for consensual attitude objects (Hypothesis 2a). The same trend was expected for social desirability (Hypothesis 2b).

The results supported both hypotheses. Regarding controversial attitude objects, namely immigration and death penalty (Experiments 1 and 2), we observed that ambivalence was evaluated significantly higher on social utility compared to univalent positive and univalent negative attitudes, in line with Hypothesis 1a. Moreover, in line with Hypothesis 1b, we observed a linear decrease from the pro-normative attitude to the ambivalent one and finally to the counter-normative one on both immigration and death penalty^2^. The pattern of result was different for consensual attitude objects, namely organic products and recycling (Experiments 3 and 4). We found the expected linear trend for both social utility and social desirability, whereby the univalent positive attitude (in both cases the pro-normative attitude) was rated higher compared to ambivalent attitude, which in turn was evaluated higher compared to univalent negative attitude. These effects were confirmed by the final small-scale meta-analysis.

These results contribute to the recent literature on attitudinal ambivalence by providing the evidence that the expression of ambivalent attitudes could be socially valued by an observer while providing insight into the mechanism responsible for this attribution of value. The present results indeed reveal that the expression of attitudinal ambivalence is recognized as competence (i.e., more socially useful) when it is expressed on a controversial attitude object. It has recently been reported that individuals could display more ambivalence toward controversial issues to gain social approval (Pillaud et al., 2013). The present research complements these findings by showing that by doing so, ambivalent individuals are evaluated as more competent compared to individuals displaying univalent attitudes (both pro- and anti-normative ones).

In fact, our results allow us to go a step further, as they show that ambivalence may indeed be perceived as bearing some social value, to the extent that it can be interpreted as a form of competence, as it is the case for attitudes regarding controversial issues. Such results are directly in line with the literature in which ambivalence has been linked to wisdom, mindfulness, and knowledge. By acknowledging both sides of controversial attitude objects, the expression of ambivalence is recognized as competence. They also help interpret the results of Costarelli (2011) and Brauer et al. (2012), who showed that the expression of ambivalence or mixed messages is considered as the most balanced, realistic, and acceptable when being expressed toward out-groups. As attitude expression toward out-groups is quite complex, mixed attitudes could then have been perceived as the most skillful solution.

This research could explain why ambivalence has been studied as something negative in the history of research on attitudes. First, if ambivalent attitudes are quite prevalent (Breckler, 2004; Weisbrode, 2012), controversies surrounding attitude objects are quite rare and episodic: although the media may depict many of the daily reported issues as controversial for marketing reasons, most people strive to hold non-controversial positions, to such an extent that Moscovici and Faucheux (1972) have termed this tendency a “conformity bias.” We thus believe that in most cases, ambivalent individuals could feel a social pressure that would push them to pick a side. Laponce (1974) and Weisbrode (2012) indeed argued that we tend to dichotomize the world and then view its parts as being opposed. Because ambivalence is associated with inconsistencies and fluctuations (Lavine, 2001; Fabrigar et al., 2005) and because consistency is valued (Guadagno and Cialdini, 2010), ambivalence toward consensual issues can therefore be seen as “abnormal.” Consequently, ambivalent individuals could be motivated not to present themselves as ambivalent and instead express univalent attitudes.

Such reasoning can also provide a better understanding of why ambivalent individuals can indeed feel conflicted. If we acknowledge that decision-making situations could generate discomfort in themselves (as van Harreveld et al., 2009b, suggested), discomfort could also stem in part from social considerations. In line with the above argument, ambivalent individuals could see themselves as deviant on consensual issues, and decision-making situations could highlight this feeling (Janis, 1972). As a reminder, Fong (2006) discussed ambivalent emotions as “unusual.” Future research should consequently address this question by assessing discomfort of ambivalent individuals in decision-making situations by accounting for the level of controversy of the attitude object. It is plausible to expect that individuals should experience more discomfort when they perceive a strong consensus on the attitude to be expressed, but less so when the attitude object is perceived as controversial.

Moreover, some target's and observer's characteristics or status might influence the appreciation of ambivalence expression. For example, as leaders who express emotional complexity induce positive inferences of cognitive flexibility from followers (Rothman and Melwani, 2017), the reciprocal status of target and observers may influence the evaluation of ambivalence expression. Furthermore, to the extent that political liberals are more likely compared to conservatives to tolerate uncertainty and ambiguity, enjoy thinking, and prolong cognitive closure (e.g., Jost et al., 2003), it follows that they should be more inclined to value others' ambivalence expression. Future research should investigate the possible moderating role of these characteristics.

This study has limitations that could open avenue for new research. In this article, we used different attitude objects, chosen after a pilot study that revealed their different level of controversy; however, future research could try to replicate this study using longitudinal methods to study how the perception of ambivalence could fluctuate as a consequence of controversies. For instance, Groenendyk (2016) highlighted that Democrats were more ambivalent toward President Obama after he reacted to Syrian president Bashar al-Assad's use of chemical weapons against his own people. It would therefore be interesting to focus on political figures or on social issues (such as gay marriage or gun control) that are more or less controversial at different time points. The study of such phenomena could represent a dynamic approach to the study of the perception of ambivalence.

In sum, this research and its results bring convergent support for viewing attitudinal ambivalence as useful. When being expressed on controversial issues, ambivalent attitudes were recognized as signaling competence over and above univalent attitudes. After more than two decades of research that has focused on its weaknesses and negative consequences, the present research might open the way to the functional study of attitudinal ambivalence.

## Ethics statement

At the time the experiments were conducted (2011–2013) neither approval by an ethics committee, nor written consent by the participants was needed in Switzerland to conduct non-medical research on human subjects. As stated by the Federal Administration of the Swiss Confederation (https://www.bag.admin.ch/bag/en/home/themen/mensch-gesundheit/biomedizin-forschung/forschung-am-menschen/entstehung-humanforschungsgesetz.html), the law relating to research on human subjects (i.e., constitutional article n°118b) came into effect in January 1st 2014. Given this legislation, the present research project was not submitted to any research ethics board.

## Author's note

This work is part of VP's doctoral thesis (Pillaud, 2013) conducted under the supervision of FB and NC and supported by the Swiss National Science Foundation. We wish to thank Nicolas Sommet for his invaluable advice on a previous version of the manuscript.

## Author contributions

VP, NC, and FB developed the study concept. VP, NC, and FB contributed to the study design. Data collection for all experiments were performed by VP. VP performed the data analysis and interpretation in collaboration with NC and FB. VP drafted the manuscript, and NC and FB provided critical revisions. All authors approved the final version of the manuscript for submission.

### Conflict of interest statement

The authors declare that the research was conducted in the absence of any commercial or financial relationships that could be construed as a potential conflict of interest.

## Footnotes

^1^It could be argued that before we move to the main studies, we also need to check one important assumption underlying our hypothesis, namely that attitudinal ambivalence may indeed signal competence, i.e., that one is critically appraising the issue. This web-based experiment was run on LimeSurvey with 146 women and 21 men (mean age 20.53 years, *SD* = 3.31). The participants were told that we were conducting a study on attitudes toward death penalty. The same procedure was used as in Experiment 1 in that three profiles displaying a positive attitude, an ambivalent attitude, and a negative attitude were presented in a random order to the participants who had to evaluate each profile. They had to evaluate each profile using six items. Two items assessed critical thinking (*critical/critique* and *thoughtful/réfléchi*; *r* = 0.62, *p* < 0.001), and we also introduced two items that assessed dissent (*deviant/déviant* and *dissenter/contestataire*; *r* = 0.24, *p* = 0.002) and two that assessed cautiousness (*careful/prudent* and *pernickety/pointilleux, r* = 0.31, *p* < 0.001) to test other possible meanings of ambivalence. We aggregated the two items per dimension and used these three indices as our dependent variables. Contrast analysis showed that the ambivalent profile has been evaluated significantly higher on *critical thinking* (*M* = 4.27) compared to the positive (*M* = 3.72) and the negative one (*M* = 4.02), *F*_(1, 166)_ = 6.45, *p* = 0.012, η^2^_*p*_ = 0.037. The orthogonal contrast did not reach significance, *F*_(1, 166)_ = 2.61, *p* > 0.10. Thus, ambivalent attitudes were rated higher on critical thinking compared to clear-cut attitudes. The contrast test of the ambivalent profile against the other two on the *dissent* dimension did not reach significance, *F*_(1, 166)_ = 3.20, *p* > 0.05. However, the orthogonal contrast yielded a significant effect, *F*_(1, 166)_ = 4.09, *p* = 0.045, indicating that the negative profile has been evaluated as less dissenting (*M* = 3.32) compared to the ambivalent (*M* = 3.63) and the positive one (*M* = 3.59). Hence, ambivalent attitudes have not been acknowledged as more deviant compared to clear-cut attitudes. We did not observe any significant differences in cautiousness as a function of the profiles for both contrasts, all *p*s > 0.10. Hence, the ambivalent profile (*M* = 3.85), the negative profile (*M* = 3.62), and the positive profile (*M* = 3.75) were rated the same.

^2^Two further experiments, not reported in full here because of their redundancy, perfectly replicated the findings of Experiments 1 and 2. Forty participants completed an experiment on repeating school years, and one-hundred participants participated in another experiment about having children, two controversial attitude objects. Performing the same contrast analyses as in Experiments 1 and 2, the results showed that the participants judged the ambivalent profile as higher on social utility compared to the univalent profiles in both studies, respectively, for repeating school years, *F*_(1, 39)_ = 4.67, *p* = 0.037, η_p_^2^ = 0.107, and for having children, *F*_(1, 99)_ = 11.26, *p* = 0.01, η_p_^2^ = 0.09. The orthogonal contrasts were not significant, all *ps* > 0.10. Concerning social desirability, the linear trend was also replicated, with the univalent pro-normative profile being evaluated higher on social desirability compared to the ambivalent profile, followed by the univalent anti-normative profile for repeating school years, *F*_(1, 39)_ = 9.99, *p* = 0.003, η_p_^2^ = 0.20, and for having children, *F*_(1, 99)_ = 139.38, *p* < 0.001, η_p_^2^ = 0.58. The orthogonal contrasts were not significant, all *ps* > 0.10. For additional information, please contact the authors.
