# Supplementary material for: The Social Utility of Ambivalence: Being Ambivalent on Controversial Issues Is Recognized as Competence
Source: Front Psychol. 2018 Jun 22;9:961. doi: 10.3389/fpsyg.2018.00961 (PMC6024988; doi:10.3389/fpsyg.2018.00961)
Supplement: Supplementary file 1 [file Table_1.docx]

**Appendix**

**Experiment 1 (Immigration)**

*Positive attitude profile*

Pas du tout Tout à fait

| 1 - Je suis favorable à la construction de minarets en Suisse.  (*I agree with the construction of minarets in Switzerland.)* | ( ) | ( ) | ( ) | ( ) | ( ) | ( ) | (x) |
| --- | --- | --- | --- | --- | --- | --- | --- |
| 2 - Je désapprouve l'interdiction du port du voile intégral dans l'espace public.  (*I disagree with the ban of full-face veils in public space*.) | ( ) | ( ) | ( ) | ( ) | ( ) | ( ) | (x) |
| 3 - Il me semble être acceptable de dispenser des élèves pour certaines branches ou activités pour des motifs religieux (comme des cours de natation ou d'éducation sexuelle par exemple).  (*It seems acceptable to exempt students for specific classes or activities for religious motives (such as swimming classes or sexual education for exemple)*. | ( ) | ( ) | ( ) | ( ) | ( ) | (x) | ( ) |
| 4 - Le renvoi des criminels étrangers ne me parait pas être une bonne solution.  (*Deporting foreign criminals does not seem a good solution to me*). | ( ) | ( ) | ( ) | ( ) | ( ) | ( ) | (x) |
| 5 - Je suis favorable à ce que le droit de vote au niveau communal soit instauré pour les étrangers qui vivent en Suisse depuis au moins 10 ans.  (*I support the establishment of a right to vote at a communal level for foreigners who have been living in Switzerland for at least ten years*)*.* | ( ) | ( ) | ( ) | ( ) | ( ) | ( ) | (x) |
| 6 - La naturalisation devrait être facilitée  (*Naturalization should be facilitated*). | ( ) | ( ) | ( ) | ( ) | ( ) | (x) | ( ) |
| 7 - Il ne me semble pas acceptable que les places de travail soient accordées prioritairement aux personnes de nationalité Suisse en temps de crise.  (*It is unacceptable that jobs are firstly attributed to Swiss citizens during times of crisis*). | ( ) | ( ) | ( ) | ( ) | ( ) | (x) | ( ) |
| 8 - Je suis favorable à propos de l'accord sur la libre circulation qui existe avec l'Union Européenne.  (*I am favourable towards the Agreement on the free movement of persons established with the European Union*) | ( ) | ( ) | ( ) | ( ) | ( ) | ( ) | (x) |

**Experiment 1 (Immigration)**

*Negative attitude profile*

Pas du tout Tout à fait

| 1 - Je suis favorable à la construction de minarets en Suisse. | (x) | ( ) | ( ) | ( ) | ( ) | ( ) | ( ) |
| --- | --- | --- | --- | --- | --- | --- | --- |
| 2 - Je désapprouve l'interdiction du port du voile intégral dans l'espace public | (x) | ( ) | ( ) | ( ) | ( ) | ( ) | ( ) |
| 3 - Il me semble être acceptable de dispenser des élèves pour certaines branches ou activités pour des motifs religieux (comme des cours de natation ou d'éducation sexuelle par exemple) | ( ) | (x) | ( ) | ( ) | ( ) | ( ) | ( ) |
| 4 - Le renvoi des criminels étrangers ne me parait pas être une bonne solution. | (x) | ( ) | ( ) | ( ) | ( ) | ( ) | ( ) |
| 5 - Je suis favorable à ce que le droit de vote au niveau communal soit instauré pour les étrangers qui vivent en Suisse depuis au moins 10 ans. | (x) | ( ) | ( ) | ( ) | ( ) | ( ) | ( ) |
| 6 - La naturalisation devrait être facilitée | ( ) | (x) | ( ) | ( ) | ( ) | ( ) | ( ) |
| 7 - Il ne me semble pas acceptable que les places de travail soient accordées prioritairement aux personnes de nationalité Suisse en temps de crise. | ( ) | (x) | ( ) | ( ) | ( ) | ( ) | ( ) |
| 8 - Je suis favorable à propos de l'accord sur la libre circulation qui existe avec l'Union Européenne | (x) | ( ) | ( ) | ( ) | ( ) | ( ) | ( ) |

**Experiment 1 (Immigration)**

*Ambivalent attitude profile*

Pas du tout Tout à fait

| 1 - Je suis favorable à la construction de minarets en Suisse. | ( ) | ( ) | ( ) | ( ) | ( ) | ( ) | (x) |
| --- | --- | --- | --- | --- | --- | --- | --- |
| 2 - Je désapprouve l'interdiction du port du voile intégral dans l'espace public | ( ) | ( ) | ( ) | ( ) | ( ) | ( ) | (x) |
| 3 - Il me semble être acceptable de dispenser des élèves pour certaines branches ou activités pour des motifs religieux (comme des cours de natation ou d'éducation sexuelle par exemple) | ( ) | ( ) | ( ) | ( ) | ( ) | (x) | ( ) |
| 4 - Le renvoi des criminels étrangers ne me parait pas être une bonne solution. | ( ) | ( ) | ( ) | ( ) | ( ) | ( ) | (x) |
| 5 - Je suis favorable à ce que le droit de vote au niveau communal soit instauré pour les étrangers qui vivent en Suisse depuis au moins 10 ans. | (x) | ( ) | ( ) | ( ) | ( ) | ( ) | ( ) |
| 6 - La naturalisation devrait être facilitée | ( ) | (x) | ( ) | ( ) | ( ) | ( ) | ( ) |
| 7 - Il ne me semble pas acceptable que les places de travail soient accordées prioritairement aux personnes de nationalité Suisse en temps de crise. | ( ) | (x) | ( ) | ( ) | ( ) | ( ) | ( ) |
| 8 - Je suis favorable à propos de l'accord sur la libre circulation qui existe avec l'Union Européenne | (x) | ( ) | ( ) | ( ) | ( ) | ( ) | ( ) |

**Experiment 2 (Death Penalty)**

*Positive attitude profile*

Pas du tout Tout à fait

| 1 - Je pense que la peine de mort permet d'éviter le risque de récidive.  *(I believe death penalty helps diminishing the risk to reoffend).* | ( ) | ( ) | ( ) | ( ) | ( ) | ( ) | (x) |
| --- | --- | --- | --- | --- | --- | --- | --- |
| 2 - La peine de mort amène un sentiment de justice aux victimes et à leurs familles.  (*Death penalty brings a feeling of justice to both victims and their families*). | ( ) | ( ) | ( ) | ( ) | ( ) | ( ) | (x) |
| 3 - Grâce à la peine de mort, on diminue le taux de criminalité.  (*Thanks to death penalty, criminality rates are diminished*). | ( ) | ( ) | ( ) | ( ) | ( ) | (x) | ( ) |
| 4 - La peine de mort dissuade les individus de commettre des crimes.  (*Death penalty deters individuals from committing crimes*) | ( ) | ( ) | ( ) | ( ) | ( ) | ( ) | (x) |
| 5 - La peine de mort coûte moins cher à la société.  (*Death penalty is less costly to society*). | ( ) | ( ) | ( ) | ( ) | ( ) | ( ) | (x) |
| 6 - Les conditions de vie des personnes détenues pour des faits mineurs peuvent être améliorées de par l'application de la peine de mort.  (*Applying death penalty could help improving the life condition of individuals convicted for minor felonies*). | ( ) | ( ) | ( ) | ( ) | ( ) | (x) | ( ) |
| 7 - La peine de mort permet de lutter contre la surpopulation carcérale.  (*Death penalty helps counter prison overcrowding*). | ( ) | ( ) | ( ) | ( ) | ( ) | (x) | ( ) |
| 8 - En exécutant les condamnés, d'avantage de moyens peuvent être investis pour la réinsertion des autres détenus.  (*By executing prisoners who have been sentenced to death, more founding can be invested in the social rehabilitation of other inmates*). | ( ) | ( ) | ( ) | ( ) | ( ) | ( ) | (x) |

**Experiment 2 (Death Penalty)**

*Negative attitude profile*

Pas du tout Tout à fait

| 1 - Je pense que la peine de mort permet d'éviter le risque de récidive | (x) | ( ) | ( ) | ( ) | ( ) | ( ) | ( ) |
| --- | --- | --- | --- | --- | --- | --- | --- |
| 2 - La peine de mort amène un sentiment de justice aux victimes et à leur famille | (x) | ( ) | ( ) | ( ) | ( ) | ( ) | ( ) |
| 3 - Grâce à la peine de mort, on diminue le taux de criminalité | ( ) | (x) | ( ) | ( ) | ( ) | ( ) | ( ) |
| 4 - La peine de mort dissuade les individus de commettre des crimes | (x) | ( ) | ( ) | ( ) | ( ) | ( ) | ( ) |
| 5 - La peine de mort coûte moins cher à la société | (x) | ( ) | ( ) | ( ) | ( ) | ( ) | ( ) |
| 6 - Les conditions de vie des personnes détenues pour des faits mineurs peuvent être améliorées de par l'application de la peine de mort | ( ) | (x) | ( ) | ( ) | ( ) | ( ) | ( ) |
| 7 - La peine de mort permet de lutter contre la surpopulation carcérale | ( ) | (x) | ( ) | ( ) | ( ) | ( ) | ( ) |
| 8 - En exécutant les condamnés, d'avantage de moyens peuvent être investis pour la réinsertion des autres détenus | (x) | ( ) | ( ) | ( ) | ( ) | ( ) | ( ) |

**Experiment 2 (Death Penalty)**

*Ambivalent attitude profile*

Pas du tout Tout à fait

| 1 - Je pense que la peine de mort permet d'éviter le risque de récidive | (x) | ( ) | ( ) | ( ) | ( ) | ( ) | ( ) |
| --- | --- | --- | --- | --- | --- | --- | --- |
| 2 - La peine de mort amène un sentiment de justice aux victimes et à leur famille | (x) | ( ) | ( ) | ( ) | ( ) | ( ) | ( ) |
| 3 - Grâce à la peine de mort, on diminue le taux de criminalité | ( ) | (x) | ( ) | ( ) | ( ) | ( ) | ( ) |
| 4 - La peine de mort dissuade les individus de commettre des crimes | (x) | ( ) | ( ) | ( ) | ( ) | ( ) | ( ) |
| 5 - La peine de mort coûte moins cher à la société | ( ) | ( ) | ( ) | ( ) | ( ) | ( ) | (x) |
| 6 - Les conditions de vie des personnes détenues pour des faits mineurs peuvent être améliorées de par l'application de la peine de mort | ( ) | ( ) | ( ) | ( ) | ( ) | (x) | ( ) |
| 7 - La peine de mort permet de lutter contre la surpopulation carcérale | ( ) | ( ) | ( ) | ( ) | ( ) | (x) | ( ) |
| 8 - En exécutant les condamnés, d'avantage de moyens peuvent être investis pour la réinsertion des autres détenus | ( ) | ( ) | ( ) | ( ) | ( ) | ( ) | (x) |

**Experiment 3 (Organic Products)**

*Positive attitude profile*

Pas du tout Tout à fait

| 1 - Les produits bio évitent l'emploi de pesticides.  (*Organic products avoids using pesticides*). | ( ) | ( ) | ( ) | ( ) | ( ) | ( ) | (x) |
| --- | --- | --- | --- | --- | --- | --- | --- |
| 2 - Les produits bio empêchent la dégradation du sol.  (*Organic products prevent soil degradation*). | ( ) | ( ) | ( ) | ( ) | ( ) | ( ) | (x) |
| 3 - Au niveau environnemental, l'agriculture bio me semble être une bonne alternative par rapport à une agriculture plus conventionnelle  (*Organic agriculture is a good alternative to more conventional agriculture at the environmental level*). | ( ) | ( ) | ( ) | ( ) | ( ) | (x) | ( ) |
| 4 - Les produits bio contribuent à un meilleur respect des ressources naturelles de la planète.  (*Organic products contribute to a better respect of planet’s natural resources*). | ( ) | ( ) | ( ) | ( ) | ( ) | ( ) | (x) |
| 5 - Les produits bio contiennent plus de vitamines et de minéraux que les produits plus conventionnels.  (*Organic products have more vitamins and minerals than more conventional products*). | ( ) | ( ) | ( ) | ( ) | ( ) | ( ) | (x) |
| 6 - Les produits bio sont meilleurs pour la santé.  (*Organic products are heatlhier*). | ( ) | ( ) | ( ) | ( ) | ( ) | (x) | ( ) |
| 7 - Les produits bio permettent de renforcer le système immunitaire.  (*Organic products help reinforcing the immune system*). | ( ) | ( ) | ( ) | ( ) | ( ) | (x) | ( ) |
| 8 – Il est sain de manger des produits bio.  (*It is wise to eat organic products*). | ( ) | ( ) | ( ) | ( ) | ( ) | ( ) | (x) |

**Experiment 3 (Organic Products)**

*Negative attitude profile*

Pas du tout Tout à fait

| 1 - Les produits bio évitent l'emploi de pesticides | (x) | ( ) | ( ) | ( ) | ( ) | ( ) | ( ) |
| --- | --- | --- | --- | --- | --- | --- | --- |
| 2 - Les produits bio empêchent la dégradation du sol | (x) | ( ) | ( ) | ( ) | ( ) | ( ) | ( ) |
| 3 - Au niveau environnemental, l'agriculture bio me semble être une bonne alternative par rapport à une agriculture plus conventionnelle | ( ) | (x) | ( ) | ( ) | ( ) | ( ) | ( ) |
| 4 - Les produits bio contribuent à un meilleur respect des ressources naturelles de la planète | (x) | ( ) | ( ) | ( ) | ( ) | ( ) | ( ) |
| 5 - Les produits bio contiennent plus de vitamines et de minéraux que les produits plus conventionnels | (x) | ( ) | ( ) | ( ) | ( ) | ( ) | ( ) |
| 6 - Les produits bio sont meilleurs pour la santé | ( ) | (x) | ( ) | ( ) | ( ) | ( ) | ( ) |
| 7 - Les produits bio permettent de renforcer le système immunitaire | ( ) | (x) | ( ) | ( ) | ( ) | ( ) | ( ) |
| 8 - Il est sain de manger des produits bio. | (x) | ( ) | ( ) | ( ) | ( ) | ( ) | ( ) |

**Experiment 3 (Organic Products)**

*Ambivalent attitude profile*

Pas du tout Tout à fait

| 1 - Les produits bio évitent l'emploi de pesticides | (x) | ( ) | ( ) | ( ) | ( ) | ( ) | ( ) |
| --- | --- | --- | --- | --- | --- | --- | --- |
| 2 - Les produits bio empêchent la dégradation du sol | (x) | ( ) | ( ) | ( ) | ( ) | ( ) | ( ) |
| 3 - Au niveau environnemental, l'agriculture bio me semble être une bonne alternative par rapport à une agriculture plus conventionnelle | ( ) | (x) | ( ) | ( ) | ( ) | ( ) | ( ) |
| 4 - Les produits bio contribuent à un meilleur respect des ressources naturelles de la planète | (x) | ( ) | ( ) | ( ) | ( ) | ( ) | ( ) |
| 5 - Les produits bio contiennent plus de vitamines et de minéraux que les produits plus conventionnels | ( ) | ( ) | ( ) | ( ) | ( ) | ( ) | (x) |
| 6 - Les produits bio sont meilleurs pour la santé | ( ) | ( ) | ( ) | ( ) | ( ) | (x) | ( ) |
| 7 - Les produits bio permettent de renforcer le système immunitaire | ( ) | ( ) | ( ) | ( ) | ( ) | (x) | ( ) |
| 8 - Il est sain de manger des produits bio. | ( ) | ( ) | ( ) | ( ) | ( ) | ( ) | (x) |

**Experiment 4 (Recycling)**

*Positive attitude profile*

Pas du tout Tout à fait

| 1 - Le recyclage permet de mieux respecter notre environnement.  (*Recycling allows a better respect of our environment*). | ( ) | ( ) | ( ) | ( ) | ( ) | ( ) | (x) |
| --- | --- | --- | --- | --- | --- | --- | --- |
| 2 - C'est grâce au recyclage et au traitement des déchets que nous pouvons économiser nos ressources naturelles.  (*It is thanks to recycling and waste treatment that we can spare our natural resources*). | ( ) | ( ) | ( ) | ( ) | ( ) | ( ) | (x) |
| 3 - Le recyclage aide à maintenir correctement notre écosystème.  (*Recycling helps maintaining our ecosystem correctly*). | ( ) | ( ) | ( ) | ( ) | ( ) | (x) | ( ) |
| 4 - Le recyclage évite de gaspiller des matériaux et des ressources naturelles.  (*Recycling prevents wasting materials and natural resources*). | ( ) | ( ) | ( ) | ( ) | ( ) | ( ) | (x) |
| 5 - Tout ce qui peut être recyclé devrait l'être, peu importe le prix auquel cela revient.  (*Anything that could be recycled should be, no matter the cost*). | ( ) | ( ) | ( ) | ( ) | ( ) | ( ) | (x) |
| 6 - Nous devrions éviter de créer de nouveaux matériaux si nous pouvons nous servir de matériaux recyclés.  (*We should avoid manufacturing new materials if we can use recycled ones*). | ( ) | ( ) | ( ) | ( ) | ( ) | (x) | ( ) |
| 7 - Quand j'ai le choix, je préfère acheter un produit recyclé qu'un produit neuf.  (*When the choice is offered, I’d rather buy a recycled product than a new one*). | ( ) | ( ) | ( ) | ( ) | ( ) | (x) | ( ) |
| 8 - Je serai favorable à ce que l'on réduise le développement de nouveaux produits afin de favoriser le recyclage.  (*I would agree on reducing the development of new products to favour recycling*). | ( ) | ( ) | ( ) | ( ) | ( ) | ( ) | (x) |

**Experiment 4 (Recycling)**

*Negative attitude profile*

Pas du tout Tout à fait

| 1 - Le recyclage permet de mieux respecter notre environnement | (x) | ( ) | ( ) | ( ) | ( ) | ( ) | (x) |
| --- | --- | --- | --- | --- | --- | --- | --- |
| 2 - C'est grâce au recyclage et au traitement des déchets que nous pouvons économiser nos ressources naturelles | (x) | ( ) | ( ) | ( ) | ( ) | ( ) | (x) |
| 3 - Le recyclage aide à maintenir correctement notre écosystème | ( ) | (x) | ( ) | ( ) | ( ) | (x) | ( ) |
| 4 - Le recyclage évite de gaspiller des matériaux et des ressources naturelles | (x) | ( ) | ( ) | ( ) | ( ) | ( ) | (x) |
| 5 - Tout ce qui peut être recyclé devrait l'être, peu importe le prix auquel cela revient | (x) | ( ) | ( ) | ( ) | ( ) | ( ) | ( ) |
| 6 - Nous devrions éviter de créer de nouveaux matériaux si nous pouvons nous servir de matériaux recyclés | ( ) | (x) | ( ) | ( ) | ( ) | ( ) | ( ) |
| 7 - Quand j'ai le choix, je préfère acheter un produit recyclé qu'un produit neuf | ( ) | (x) | ( ) | ( ) | ( ) | ( ) | ( ) |
| 8 - Je serai favorable à ce que l'on réduise le développement de nouveaux produits afin de favoriser le recyclage | (x) | ( ) | ( ) | ( ) | ( ) | ( ) | ( ) |

**Experiment 4 (Recycling)**

*Ambivalent attitude profile*

Pas du tout Tout à fait

| 1 - Le recyclage permet de mieux respecter notre environnement | ( ) | ( ) | ( ) | ( ) | ( ) | ( ) | (x) |
| --- | --- | --- | --- | --- | --- | --- | --- |
| 2 - C'est grâce au recyclage et au traitement des déchets que nous pouvons économiser nos ressources naturelles | ( ) | ( ) | ( ) | ( ) | ( ) | ( ) | (x) |
| 3 - Le recyclage aide à maintenir correctement notre écosystème | ( ) | ( ) | ( ) | ( ) | ( ) | (x) | ( ) |
| 4 - Le recyclage évite de gaspiller des matériaux et des ressources naturelles | ( ) | ( ) | ( ) | ( ) | ( ) | ( ) | (x) |
| 5 - Tout ce qui peut être recyclé devrait l'être, peu importe le prix auquel cela revient | (x) | ( ) | ( ) | ( ) | ( ) | ( ) | ( ) |
| 6 - Nous devrions éviter de créer de nouveaux matériaux si nous pouvons nous servir de matériaux recyclés | ( ) | (x) | ( ) | ( ) | ( ) | ( ) | ( ) |
| 7 - Quand j'ai le choix, je préfère acheter un produit recyclé qu'un produit neuf | ( ) | (x) | ( ) | ( ) | ( ) | ( ) | ( ) |
| 8 - Je serai favorable à ce que l'on réduise le développement de nouveaux produits afin de favoriser le recyclage | (x) | ( ) | ( ) | ( ) | ( ) | ( ) | ( ) |
